# Population aging and divergent burden trajectories of fall-related spinal cord injury: a cross national analysis

**DOI:** 10.3389/ijph.2026.1609458

**Published:** 2026-07-14

**Authors:** Kong-Jun Yuan, Dan Zhu, Zeng-Jin Huang, Jia-Ming Zhong, Si-Xiong Shi, Lin Lin, Ji-Xiang Zhou, Gen-Long Jiao, Shuai Zheng

**Affiliations:** Dongguan Key Laboratory of Central Nervous System Injury and Repair/Dongguan Institute of Spine and Spinal Cord Injury, The Sixth Affiliated Hospital of Jinan University (Dongguan), Dongguan, China

**Keywords:** age-period-cohort analysis, cross-country comparison, decomposition analysis, disease burden, falls

## Abstract

**Objectives:**

To examine how population aging influences the burden of fall-related spinal cord injury (SCI) across countries at different demographic stages.

**Methods:**

Using GBD 2023 data, we analyzed age-standardized incidence, prevalence, and years lived with disability (YLD) rates of fall-related SCI in India, China, the United States, and Japan. Trends and drivers were assessed using EAPC, joinpoint regression, age-period-cohort models, and demographic decomposition.

**Results:**

China showed a significant increase in incidence (AAPC = 1.62%, 95% CI: 0.89–2.36) with a rise-peak-decline-rebound pattern. The United States maintained stable incidence but achieved a marked reduction in YLD burden (AAPC = −2.58%, 95% CI: −3.13 to −2.03). India and Japan exhibited lower burdens and sustained improvements; India’s YLD rate declined by 1.58% annually (95% CI: −1.93 to −1.22). APC analysis identified an age-progressive risk pattern in China but age-regressive patterns in the United States and India. Population aging was the main driver of increased prevalence in China (39.53%).

**Conclusion:**

Fall-related SCI burden varies across demographic stages. Population aging is a key determinant in aging societies, highlighting the need for stage-specific prevention and care strategies.

## Introduction

SCI is a devastating neurological disorder, leading to irreversible impairment of sensory, motor, and autonomic functions and imposing a profound global burden on both quality of life and healthcare economies [1–3]. The Global Burden of Disease (GBD) study reported over 20 million prevalent cases of SCI in 2019, with approximately 900,000 new cases annually contributing to 6.2 million years lived with disability (YLDs) [4]. The economic impact is staggering. Lifetime costs per patient are estimated at US$ 1.5-3 million in high-income countries, and global unemployment rates among adults with SCI exceed 60% [5]. This challenge is further intensified by global population aging, which has been reshaping the epidemiological profile of SCI: in many high- and middle-income regions, falls have overtaken road traffic accidents as the leading traumatic etiology [6, 7]. Older adults are at particularly high risk due to age-related osteoporosis, balance decline, and comorbidities [8]. Consequently, the absolute burden of fall-related SCI is projected to rise in tandem with population aging, even if age-standardized incidence rates(ASIR) remain stable.

Current management of SCI, including surgical [8–10] and non-surgical approaches [11], remains largely palliative rather than restorative, highlighting the importance of primary prevention. A population-level understanding of fall-related SCI is therefore essential for informing prevention strategies. Although population aging is a global phenomenon, the burden of fall-related SCI varies considerably across countries, reflecting differences in demographic transition and health system capacity [5]. However, cross-national studies examining fall-related SCI from the perspective of population aging remain limited. To address this gap, we investigated fall-related SCI at the intersection of demographic transition and population aging. India, China, the United States, and Japan were selected to represent a demographic continuum from a pre-aging society to a prototypical super-aged society. These countries encompass distinct aging trajectories and health system contexts, providing a quasi-experimental framework to explore the contribution of population aging to fall-related SCI burden while minimizing other structural influences [12–14]. We hypothesized that these demographic and systemic differences would be reflected in distinct national patterns of fall-related SCI incidence, outcomes, and overall burden.

Using GBD 2023 data, this study pursues three objectives: (1) compare epidemiological trends of fall-related SCI across four countries during 2004–2023; (2) employ age-period-cohort modeling to explore temporal factors behind prevalence trends; (3) decompose disease burden changes into contributions from population growth, aging and epidemiological shifts. Combined methods elucidate how demographic structure, especially population aging, causes cross-national disparities in fall-related SCI burden. The findings lay an evidence base for context-specific public health strategies tailored to countries with distinct demographic and health system transitions.

## Methods

### Data source and processing

Data on incidence, prevalence and YLDs of fall-related SCI were retrieved from the 2023 Global Burden of Disease (GBD 2023) for China, India, Japan, the United States and the global average [15]. Covering 375 diseases and injuries across 204 countries/territories from 1990 to 2023, all estimates including 95% uncertainty intervals (UIs) were acquired via the Global Health Data Exchange tool (https://vizhub.healthdata.org/gbd-results/). We extracted data with settings: cause as spinal cord injury, risk factor as falls, and metrics as incidence, prevalence and YLDs. All rates were age-standardized per 100,000 population based on the GBD global standard population.

GBD 2023 integrated over 310,000 data sources via standardized models. It linked ICD codes for SCI (ICD-10: S14, S24, S34, T06.0; ICD-9: 806, 952) and falls (ICD-10: W00-W19; ICD-9: E880-E888) to confirm their causal association, and classified SCI into neck-level and below-neck-level subtypes differing in clinical severity. Estimates were produced using Bayesian models including DisMod-MR 2.1 and comparative risk assessment, stratified by age, sex, region and year with 95% UIs. Strict data cleaning, outlier screening, model imputation, cross-validation and sensitivity analyses were adopted to guarantee reliable results [16].

### Descriptive trends and estimated annual percentage change (EAPC)

To quantify the overall linear trend from 2004 to 2023, we calculated the EAPC for each age-standardized rate (ASR). This was achieved by fitting a linear regression model to the natural logarithm of the annual ASR:

$$\ln\left(\text{ASR}\right)=\alpha +\beta \times \text{year}+\varepsilon$$

Where β represents the slope. The EAPC and its 95% confidence interval (CI) were derived as 100×(e^β^-1). A trend was deemed statistically significant if the 95% CI of the EAPC did not include zero.

### Joinpoint regression analysis

To detect significant inflection points and temporal phases of trends, we conducted joinpoint regression using the National Cancer Institute’s Joinpoint Regression Program (Version 5.0.2) [17]. This iterative algorithm determines the optimal number of valid joinpoints and divides time series into linear segments, with a maximum of 3 joinpoints allowed. The optimal model was selected based on the Bayesian Information Criterion (BIC). The model estimates segmental annual percentage change (APC) and overall average annual percentage change (AAPC), a weighted average of individual APCs. Results were regarded as statistically significant if the 95% CI excluded zero.

### Age-period-cohort modelling

An age-period-cohort model was built to explore trend drivers. The intrinsic estimator method was adopted to address the inherent identifiability issue of the model [18, 19]. This method applies constraints orthogonal to the design matrix null vector to eliminate bias from reference category selection, and decomposes age, period and cohort effects into orthogonal components free of arbitrary parameterization [20]. Data were grouped into 5-year age bands (<5 to 95+ years) and 5-year calendar periods (2004–2008 to 2019–2023) to define corresponding birth cohorts.

The model assumes Poisson-distributed case counts and adopts a log-linear equation. Coefficients of period and cohort effects were exponentiated to calculate risk ratios relative to the overall mean, and age effect coefficients were exponentiated to obtain age-specific rate ratios. Analyses were conducted using the apc package in R (version 4.3.1).

### Demographic decomposition analysis

Demographic decomposition analysis was conducted to quantify drivers of temporal changes in fall-related SCI burden. Total variations in incidence, prevalence and YLD case counts from 2004 to 2023 were decomposed into three factors: population growth, population aging, and shifts in age-specific epidemiological rates. A Kitagawa-based approach was applied for exact additive decomposition of case count differences between the 2 years. Each factor’s contribution was assessed via counterfactual analyses: one factor was adjusted to its 2023 level while the other two remained at 2004 values. The total case change ΔY was split as shown in the formula.

ΔY=ΔY population+ΔY aging+ΔY rates

Positive values indicate increased disease burden, while negative values denote reduced burden. This method enables accurate attribution of overall burden changes to demographic and epidemiological factors.

### Ethical considerations and reproducibility

This study used aggregated, de-identified public data from GBD, so ethics approval and informed consent were waived. Raw data are accessible via the Global Health Data Exchange. Analytical code can be obtained from the corresponding author upon reasonable request.

## Results

### National trends in the burden of fall-related spinal cord injury (2004–2023)

Cross-country comparisons revealed substantial disparities in the burden of fall-related SCI in 2023 (Supplementary Material 1). The highest ASIRs were observed in the United States (13.78 per 100,000; 95% UI: 7.98–24.26) and China (12.61; 7.79–20.23), both markedly exceeding the global average (6.93; 4.32–11.32). China also recorded the highest ASPR (359.00; 308.05–410.74) and ASYR (99.32; 66.97–132.15), followed by the United States, whereas India and Japan consistently exhibited the lowest burden. In absolute terms, China accounted for approximately 185,462 incident cases, 6.44 million prevalent cases, and 1.75 million YLDs in 2023. From 2004 to 2023, India showed the greatest declines across all burden indicators, while Japan and the United States also experienced significant reductions. In contrast, China exhibited slight increases in ASIR (EAPC = 0.84%) and ASPR (EAPC = 0.50%), with ASYR remaining relatively stable (EAPC = −0.16%).

**FIGURE 1:**
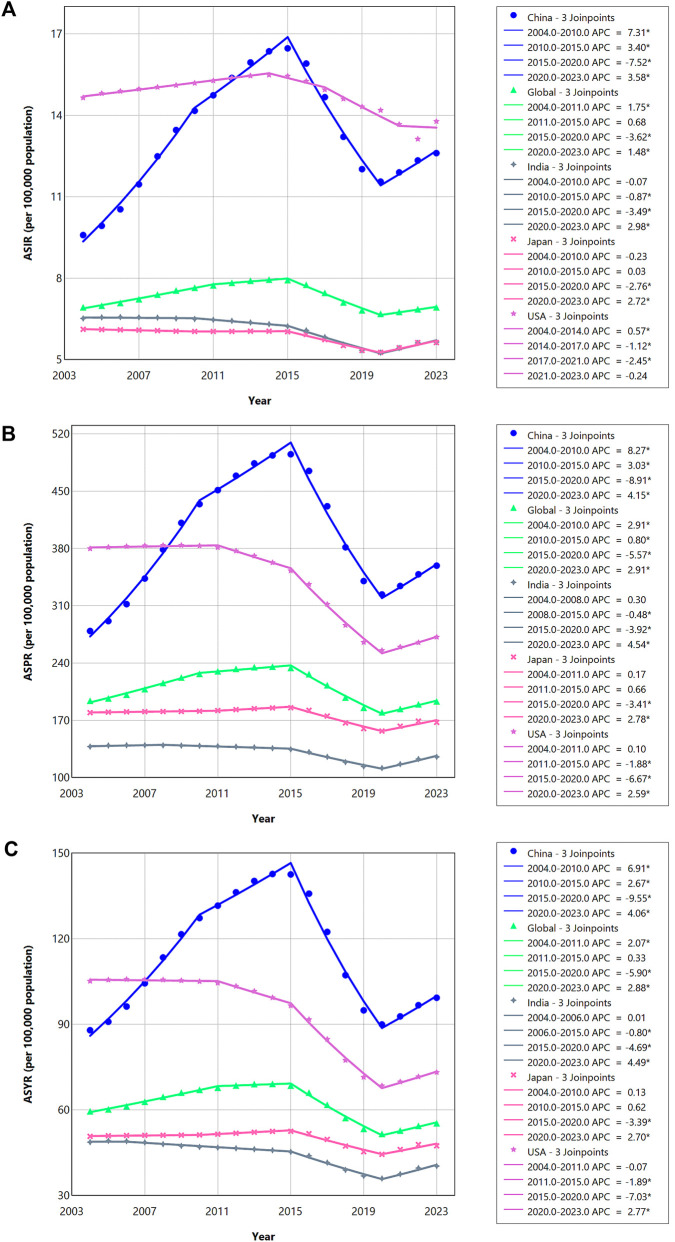
Joinpoint regression analysis of the age-standardized incidence rates, age-standardized prevalence rates, and age-standardized years lived with disability rates for fall-related spinal cord injuries. Note: Different colored lines represent age-standardized rates for each country. *Indicates that the annual percentage change in this segment is statistically significant. (Population aging and divergent burden trajectories of fall-related spinal cord injury: a cross national analysis, China, India, Japan, and the United States, 2004-2023).

Joinpoint regression revealed considerable non-linear variation in these trends (Figure 1; Supplementary Materials 2, 3). Most countries followed a three-phase pattern characterized by stability or increase before 2015, decline through 2020, and subsequent rebound. China showed the largest fluctuations, with all indicators peaking around 2015. ASIR increased rapidly during 2004–2010 (APC = 7.31%), declined during 2015–2020 (APC = −7.52%), and rose again thereafter (APC = 3.58%), resulting in a significant overall increase (AAPC = 1.62%). By contrast, the United States maintained relatively stable incidence while achieving marked reductions in prevalence (AAPC = −2.42%) and disability burden (AAPC = −2.58%). India and Japan demonstrated sustained improvements across all burden measures throughout the study period.

**FIGURE 2:**
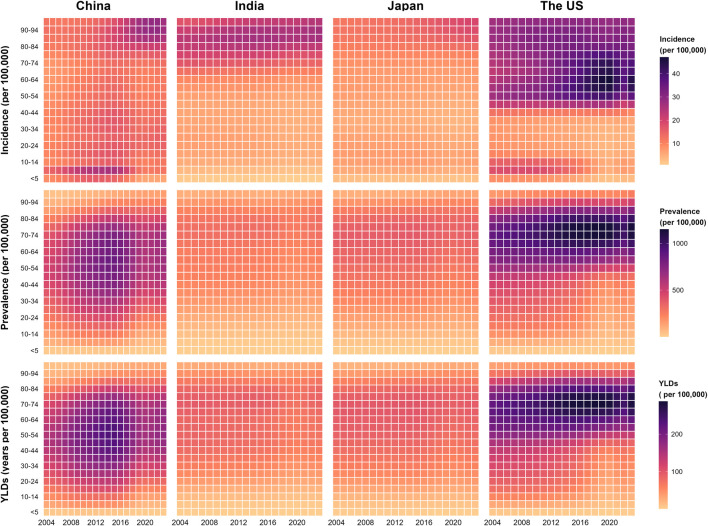
Heatmap of incidence, prevalence, and years lived with disability across different age groups in four countries from 2004 to 2023. Darker colors represent higher disease burden per 100,000 population. (Population aging and divergent burden trajectories of fall-related spinal cord injury: a cross national analysis, China, India, Japan, and the United States, 2004-2023).

Age-stratified analyses showed that incidence, prevalence, and YLDs generally increased with age, although the magnitude varied across countries (Figure 2). China and the United States exhibited the steepest age-related increases, whereas India and Japan showed more gradual age gradients. China displayed a rise-peak-decline-rebound pattern across age groups, while the United States showed declining burdens below age 50 but increasing burdens among older adults. Males consistently experienced higher burdens than females, and cervical injuries contributed more to disability burden than injuries below the neck. Across sex and injury subgroups, China and the United States consistently exhibited the highest burdens, whereas India and Japan had the lowest (Supplementary Material 4–6).

### Age-period-cohort analysis of fall-related SCI burden

**FIGURE 3:**
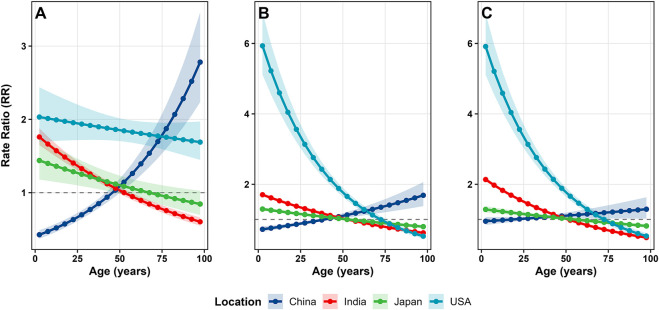
Age rate ratios of incidence **(A)**, prevalence **(B)**, and years lived with disability **(C)** of fall-related spinal cord injuries (China, India, Japan, and the United States, 2004–2023).

Age effects revealed substantial heterogeneity in life-course risk profiles across countries (Figure 3). Prevalence and YLDs exhibited age patterns largely consistent with incidence within each country. China showed a pronounced age-progressive pattern, with incidence RR increasing from 0.43 (95% CI: 0.37–0.49) in the youngest age group to 2.78 (95% CI: 2.24–3.46) among those aged 95–99 years, indicating aging as the dominant risk factor. In contrast, India and the United States displayed age-regressive profiles, with incidence RR declining from 1.76 (1.65–1.88) to 0.60 (0.55–0.66) in India and from 2.03 (1.69–2.44) to 1.69 (1.45–1.97) in the United States. The United States also exhibited the steepest age gradient in prevalence, declining from 5.93 (5.12–6.88) to 0.53 (0.46–0.60). Japan demonstrated an intermediate pattern, with elevated risk persisting through mid-adulthood before gradually approaching baseline levels in older age groups.

**FIGURE 4:**
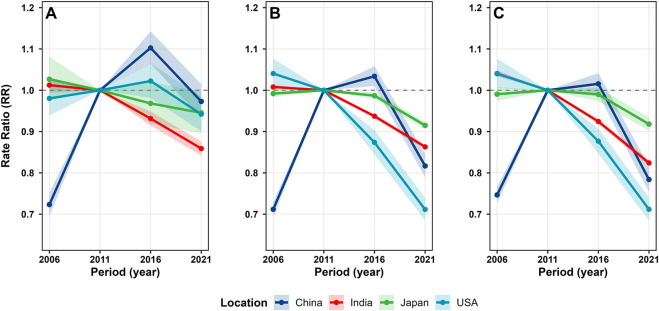
Period rate ratios of incidence **(A)**, prevalence **(B)**, and years lived with disability **(C)** of fall-related spinal cord injuries. (Population aging and divergent burden trajectories of fall-related spinal cord injury: a cross national analysis, China, India, Japan, and the United States, 2004-2023).

Period effects demonstrated contrasting temporal dynamics among countries (Figure 4). China exhibited a pronounced inverted-V pattern across all burden metrics, with incidence RR increasing from 0.72 (0.70–0.75) in 2004–2008 to 1.10 (95% CI:1.06–1.14) in 2014–2018 before declining to 0.97 (0.93–1.01) in 2019–2023. Similar trajectories were observed for prevalence and YLDs. By contrast, India, Japan, and the United States experienced sustained reductions in period-related risk. The decline was most pronounced in the United States, where prevalence and YLD RRs decreased to 0.71, representing reductions exceeding 30%. India showed steady improvements across all metrics, whereas Japan experienced more gradual declines throughout the study period.

**FIGURE 5:**
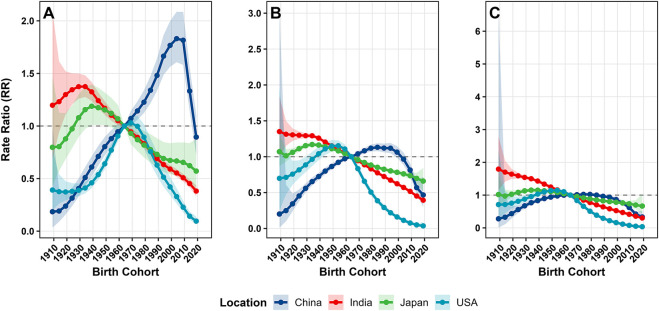
Birth cohort rate ratios of incidence **(A)**, prevalence **(B)**, and years lived with disability **(C)** of fall-related spinal cord injuries. (Population aging and divergent burden trajectories of fall-related spinal cord injury: a cross national analysis, China, India, Japan, and the United States, 2004-2023).

Cohort effects identified two distinct generational patterns (Figure 5). China and the United States exhibited unimodal trajectories characterized by an initial rise followed by a decline. In China, incidence RR increased from 0.19 (95% CI: 0.04–0.98) in the earliest birth cohort to a peak of 1.83 (1.61–2.09) around the 2004 cohort before falling to 0.89 (0.72–1.11) in the most recent cohort. The United States showed a similar pattern but peaked earlier and at a lower level, followed by a marked decline to 0.10 (0.07–0.14) in the youngest cohort. In contrast, India and Japan demonstrated sustained generational improvements after an early high-risk plateau. Incidence RR in India declined from approximately 1.38 among cohorts born between 1929 and 1939 to 0.38 (0.33–0.44), while Japan followed a comparable but less pronounced trajectory. Prevalence and YLD cohort effects closely mirrored the incidence patterns. Overall, cohort trends suggest a concentrated generational burden in China and the United States, but progressive attenuation of risk across successive generations in India and Japan.

### Decomposition analysis of drivers underlying changes in disease burden

**FIGURE 6:**
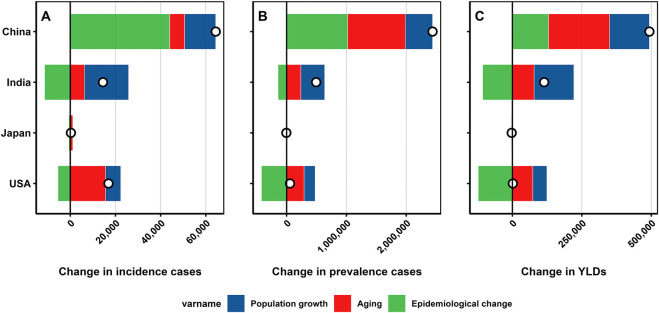
Changes in incidence **(A)**, prevalence **(B)**, and years lived with disability **(C)** of fall-related spinal cord injuries driven by population growth (blue), population aging (red) and epidemiological variation (green). Positive values on the right of zero indicate increased disease burden, and white circles represent the overall total change. (Population aging and divergent burden trajectories of fall-related spinal cord injury: a cross national analysis, China, India, Japan, and the United States, 2004-2023).

Decomposition analysis revealed distinct drivers of changes in fall-related SCI burden between 2004 and 2023 (Figure 6, Supplementary Material 7). In China, epidemiological change was the primary driver of increased incident cases (+64,385; +53.18%), accounting for 68.37% of growth, whereas population aging contributed most to increases in prevalence (+61.07%) and YLDs (+39.10%), accounting for 39.53% and 44.55% of growth, respectively. In India, population growth was the dominant positive contributor across all burden measures, but its effect was largely offset by favorable epidemiological changes, particularly for incident cases (−78.45%). In Japan and the United States, demographic and epidemiological forces acted in opposite directions. Although population aging strongly increased incident cases (383.79% in Japan and 92.18% in the United States), improvements in age-standardized rates partially offset these effects. Consequently, Japan experienced declines in prevalent cases (−1.62%) and YLDs (−2.97%), whereas the United States showed modest increases in prevalence (+4.41%) and YLDs (+0.44%). Detailed results are presented in Supplementary Material 8.

## Discussion

The four countries represent distinct stages of population aging, and this framing reveals patterns not evident from country-by-country description. At the pre-aging extreme (India), age-standardized rates (ASRs) remain low, yet absolute burden continues to rise due to rapid population growth. This suggests a critical window for proactive primary prevention to avoid future aging-related accumulation of burden [14]. Although modest ASR improvements likely reflect early gains from primary healthcare expansion, these effects remain vulnerable to demographic pressure. In aging societies (China and the United States), both experience aging-related pressure but with divergent outcomes. China’s volatile trajectory may reflect rapid industrialization and urbanization that increased exposure to risk, while preventive and regulatory systems lagged behind. Limited community-based prevention and rehabilitation capacity may have further weakened continuity of care, with a gap between acute treatment and long-term management potentially contributing to accumulated disability [10, 20]. In contrast, the United States shows stable incidence but declining disability burden, consistent with strong post-acute rehabilitation and long-term care systems [9, 13]. Although persistent incidence indicates remaining gaps in upstream prevention. At the super-aged end (Japan), burden remains low and stable, likely reflecting integrated long-term care systems, community-based care structures, and age-friendly environments that buffer aging-related risk accumulation [12]. Overall, aging does not deterministically increase burden; rather, health system integration across prevention, acute care, and long-term support appears to modulate its impact [21, 22].

Subgroup patterns further indicate that systemic disparities translate into heterogeneous risk distributions. Higher male burden may reflect incomplete enforcement of occupational safety protections in high-risk industries such as construction and manufacturing [23, 24]. Age and sex specific injury patterns suggest differentiated prevention needs, with thoracolumbar injuries more common in younger adults and cervical injuries in older populations. This supports targeted strategies such as occupational protection and safety training for younger workers, and fall prevention through strength training and environmental modification for older adults [25]. Prior evidence indicates structured exercise programs can reduce fall risk by approximately 28%, supporting their preventive value [26, 27]. The steepness of age-burden curves may also reflect system preparedness, with China’s sharper increase suggesting gaps in community and long-term care, while Japan’s flatter pattern is consistent with more effective integrated aging-care systems [12, 28].

Age-period-cohort decomposition further suggests that observed trends are shaped by interacting temporal dimensions. Age effects vary markedly: China shows a strong age-progressive pattern consistent with physiological aging and insufficient environmental adaptation [29–31], whereas the United States and India show more age-regressive patterns likely driven by occupational and behavioral exposures in younger populations [12, 28]. Conversely, the age-regressive patterns observed in the US and India point to socio-structural risks, albeit with different etiologies. India’s pattern may be related to a large young workforce concentrated in high-risk industries with weak safety protections, while the US pattern is more plausibly associated with high-risk behavioral and recreational exposures [32]. Japan demonstrates an intermediate pattern, potentially reflecting life-course safety regulation combined with structured aging-care interventions. Period effects indicate an inverted V-shaped fluctuation in China, possibly reflecting rapid risk expansion followed by partial control efforts, while other countries show more stable declines consistent with institutionalized prevention [33–36]. A synchronous decline across countries around 2015–2020 may reflect global improvements in trauma care or data systems, while subsequent divergence highlights the importance of domestic health system structure. Cohort effects suggest historical exposure patterns, with earlier high-risk cohorts gradually replaced by cohorts benefiting from improved safety, healthcare access, and education, implying that current burden partly reflects past exposures rather than only contemporary risk [37].

Demographic decomposition further clarifies that China’s burden is increasingly driven by population aging rather than epidemiological deterioration, suggesting a need to shift toward integrated community rehabilitation and long-term care systems. In the United States and Japan, epidemiological improvement partially offsets aging, but only Japan achieves net reduction, likely due to population decline combined with integrated care infrastructure [9, 13]. In contrast, continued population growth in the United States sustains total burden despite clinical and rehabilitation advances. India’s trajectory is dominated by population expansion, indicating that rapid scaling of primary prevention and trauma care systems is essential to prevent future escalation.

### Conclusion

This study examined the burden of fall-related SCI in China, the United States, Japan, and India from 2004 to 2023 using age-period-cohort and demographic decomposition analyses. The findings reveal substantial cross-country heterogeneity, largely reflecting differences in demographic transitions and health system responses. While aging and population growth remain key drivers of burden, their impacts vary according to national capacities for prevention, treatment, rehabilitation, and long-term care. Overall, the results underscore the importance of integrated, life-course approaches to injury prevention and management, and provide evidence to support context-specific strategies for reducing the burden of fall-related SCI in diverse demographic settings.
