# Supplementary material for: Population aging and divergent burden trajectories of fall-related spinal cord injury: a cross national analysis
Source: Int J Public Health. 2026 Jul 14;71:1609458. doi: 10.3389/ijph.2026.1609458 (PMC13407379; doi:10.3389/ijph.2026.1609458)
Supplement: Supplementary file 1 [file DataSheet1.docx]

**Divergent Trajectories of Fall-Related Spinal Cord Injury Burden in China, the United States, Japan, and India: An Age-Period-Cohort and Decomposition Analysis**

***Sex and Lesion-Level Disparities***

Beyond national aggregates, the burden of fall-related SCI exhibited marked disparities by sex and anatomical level of injury (Supplementary File 4). A consistent male preponderance was evident across all four nations and for all three burden metrics in 2023. Globally, the ASIR for neck-level injuries in males (4.82 per 100,000) was approximately double that in females (2.45 per 100,000), a ratio that held for injuries below the neck level.

The distribution of burden between neck-level and below-neck-level lesions further revealed distinct, sex-specific patterns. In China, the ASIR for below-neck lesions in males (8.14 per 100,000) surpassed that for neck-level injuries a reversal not seen in females. Conversely, at the global level, below-neck lesions constituted a larger proportion of total prevalence in females, while the male burden was nearly equivalent between the two injury levels. The disability burden, measured by ASYR, was consistently higher for neck-level injuries in both sexes, with a more pronounced gradient in males.

These sex-based disparities were superimposed on persistent national disparities. The United States and China consistently presented the highest burden for both sexes across all metrics, whereas Japan and India recorded the lowest. Notably, the long-term trends for both injury levels closely mirrored the overall national trends described above (detailed trends are provided in Supplementary File 5 and Supplementary File 6). The sharpest declines in the burden of below-neck lesions occurred in the United States and India.

Supplementary File 1 Incidence, prevalence, and years lived with disability of fall-related spinal cord injuries in 2023, together with estimated annual percentage changes of age-standardized rates. (China, India, Japan, and the United States, 2004-2023)

Abbreviations: ASR, age-standardized rate. EAPC, estimated annual percentage change. UI, uncertainty interval. CI, confidence interval. YLDs, years lived with disability.*Note: AAPC is statistically significant (95 CI does not include zero).

Supplementary File 2 Joinpoint regression analysis of age-standardized rates and average annual percentage changes for fall-related spinal cord injuries (China, India, Japan, and the United States, 2004-2023)

Abbreviations: AAPC, average annual percentage change. ASIR, age-standardized incidence rate. ASPR, age standardized prevalence rate. ASYR, age-standardized years lived with disability rate. CI, confidence interval.*Note: AAPC is statistically significant (95 CI does not include zero).

Supplementary File 3 Annual percentage changes and segmented time intervals identified by joinpoint regression for age-standardized rates of fall-related spinal cord injuries. (China, India, Japan, and the United States,2004-2023)

Note: * Indicates that the APC for that segment is statistically significant (95% CI does not include zero).

**
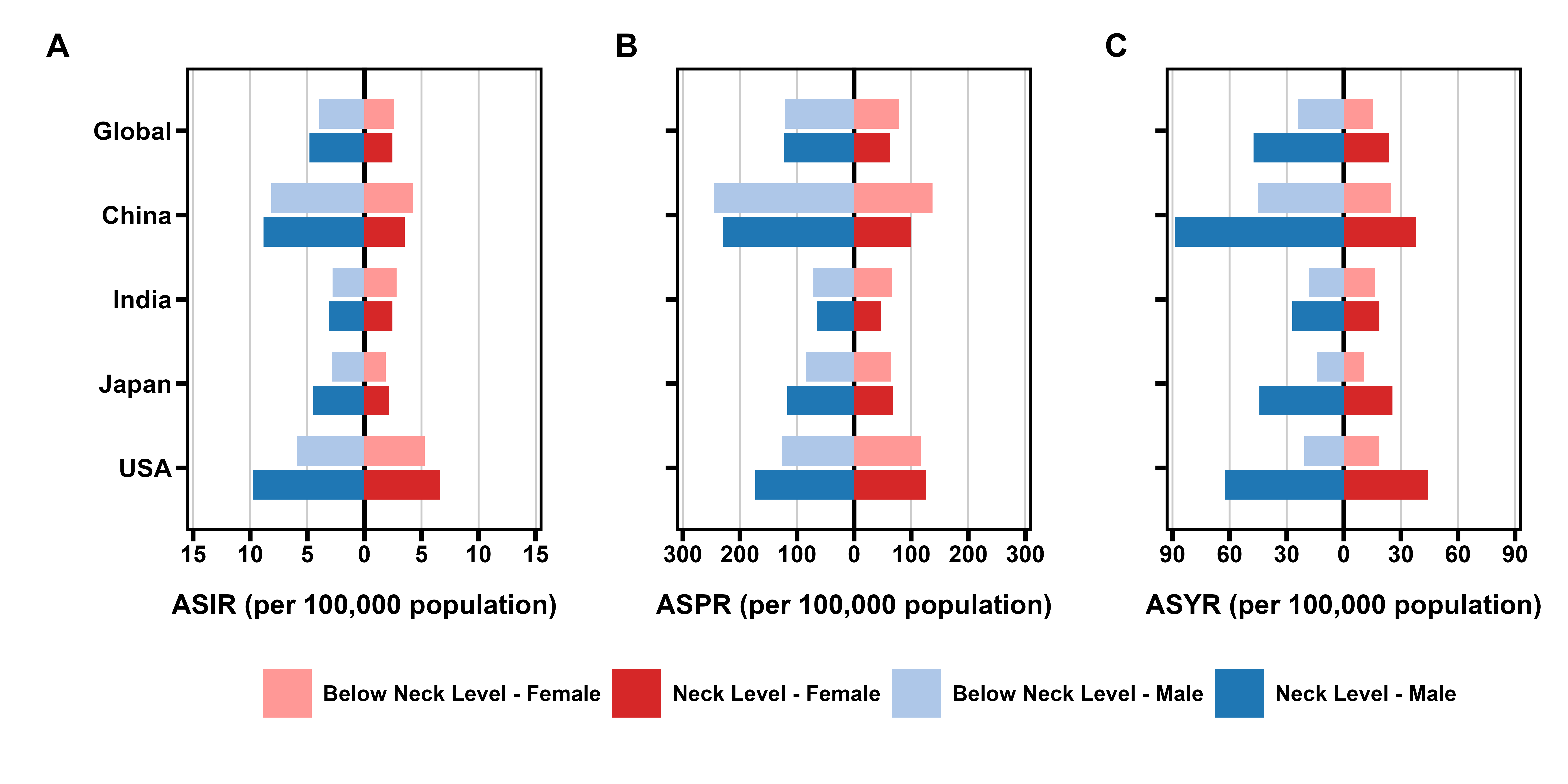
**

Supplementary File 4 Age-standardized rates of fall-related spinal cord injuries at the neck level and below the neck level among males (left) and females (right) globally and in four countries in 2023. (A) Age-standardized incidence rate, (B) age-standardized prevalence rate, (C) age-standardized years lived with disability rate. (China, India, Japan, and the United States, 2004-2023)


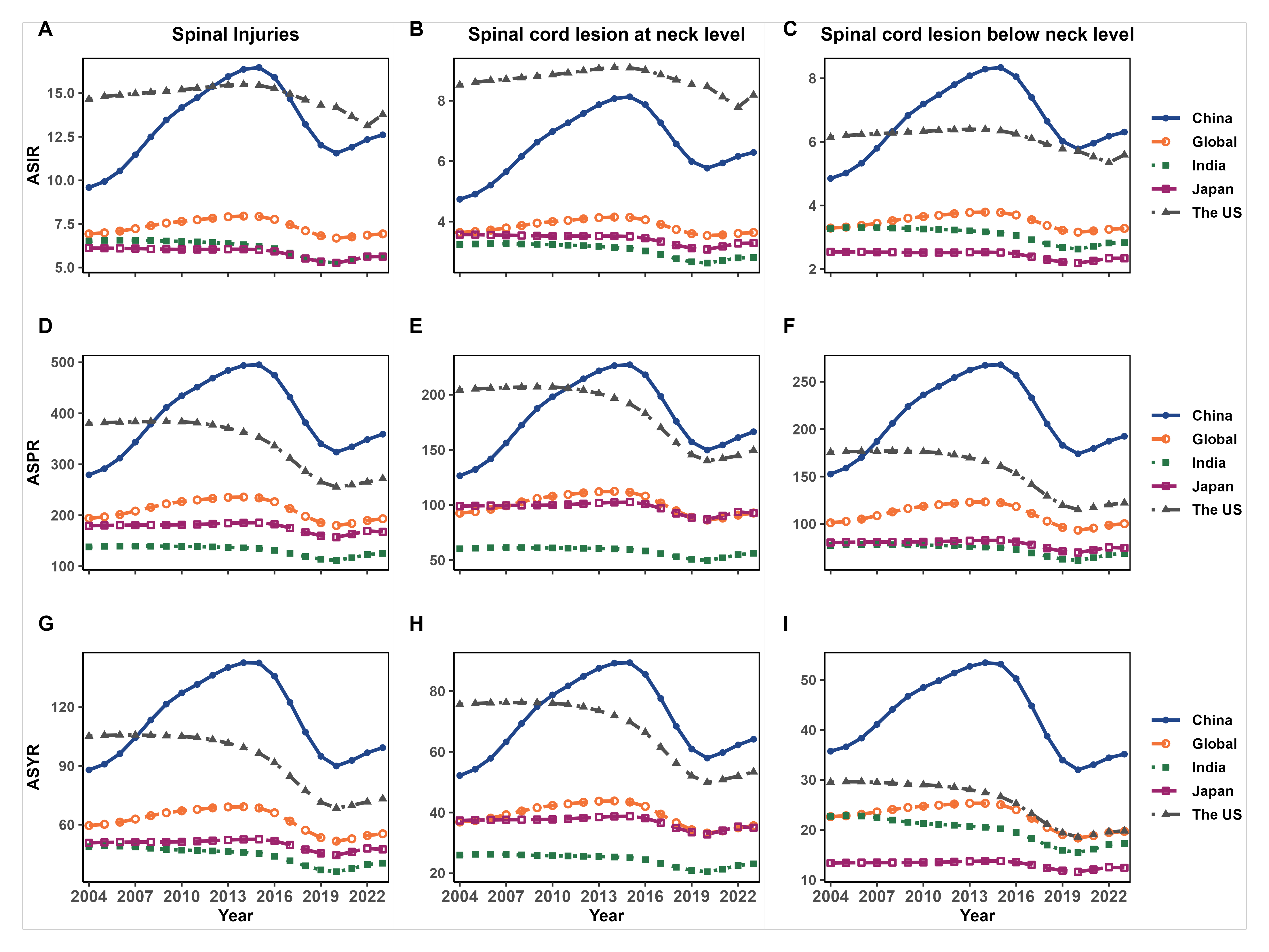


Supplementary File 5 Age-standardized rate trends for fall-related spinal cord injuries across four countries and the global level, 2004–2023. (A-C) Incidence, (D-F) Prevalence, (G-I) Years lived with disability. (China, India, Japan, and the United States, 2004-2023)

Supplementary File 6 Incidence, prevalence, and years lived with disability of fall-related spinal cord injuries stratified by lesion level in 2023, as well as estimated annual percentage changes of age-standardized rates. (China, India, Japan, and the United States, 2004-2023)

Abbreviations:ASR, age-standardized rate. EAPC, estimated annual percentage change. UI, uncertainty interval. CI, confidence interval; YLDs, years lived with disability.Note: An asterisk () denotes an EAPC whose 95% CI does not include zero (statistically significant trend).*

Supplementary File 7 Key results of demographic decomposition analysis. (China, India, Japan, and the United States, 2004-2023)

Notes: Total Change (%) and ASR Change (%) were derived from the decomposition analysis. The Leading Driver denotes the component with the largest absolute contribution to overall change. For prevalence and YLDs in Japan, the component with the largest negative contribution is reported as the Leading Negative Driver. Detailed decomposition results are presented in Supplementary File 8.

Supplementary File 8 Complete Numerical Results of the Demographic Decomposition Analysis.(China, India, Japan, and the United States, 2004-2023)
